# YouTube Videos to Create a “Virtual Hospital Experience” for Hip and Knee Replacement Patients to Decrease Preoperative Anxiety: A Randomized Trial

**DOI:** 10.2196/ijmr.4295

**Published:** 2016-04-18

**Authors:** Mary I O'Connor, Katharyn Brennan, Shari Kazmerchak, Jason Pratt

**Affiliations:** ^1^ Center for Musculoskeletal Care Department of Orthopaedics and Rehabilitation Yale School of Medicine New Haven, CT United States; ^2^ Mayo Clinic Department of Orthopedic Surgery Jacksonville, FL United States; ^3^ Mayo Clinic Center for Social Media Jacksonville, FL United States

**Keywords:** hip arthroplasty, hip replacement, knee arthroplasty, knee replacement, preoperative anxiety, virtual hospital experience, YouTube videos

## Abstract

**Background:**

With declining reimbursement to health care systems, face-to-face time between patients and providers to optimize preoperative education and counseling may be challenging.

**Objective:**

Because high patient anxiety prior to surgery has been linked to more severe and persistent pain after joint replacement surgery, the Orthopedic Surgery Department at Mayo Clinic in Florida created a playlist of 16 YouTube videos aimed at creating a virtual hospital experience for primary total hip and knee joint replacement patients. A randomized trial was then performed to evaluate the potential impact of viewing this playlist on preoperative anxiety.

**Methods:**

Each patient completed a Generalized Anxiety Disorder (GAD) score assessment at the time of the routine preoperative clinic visit and then randomized based on his/her gender, type of surgery, and initial GAD score to either the control group of standard education (education at face-to-face clinical visits as well as printed educational materials) or the treatment group (standard education plus access to the YouTube playlist). On the morning of the patient’s surgery, the same survey was repeated. Of the 65 patients who consented to participate in the study, 53 completed the study (82%) with 28 of 29 (97% completed) in the control group and 25 of 36 (69% completed) in the treatment group.

**Results:**

Overall, the results showed a trend toward less anxiety in patients who viewed the YouTube videos; this was exhibited by a reduction in the median GAD score by 1 point. This trend is more clearly present in patients with high preoperative anxiety (predominantly women), as seen in the reduction of the median GAD score by 6 points in the treatment group.

**Conclusions:**

Although our experience is limited, our results indicate that a series of tailored videos may decrease patient anxiety preoperatively. We recommend further exploration of both this concept and the use of social media tools in preoperative patient education.

**Trial Registration:**

Clinicaltrials.gov NCT02546180; http://clinicaltrials.gov/ct2/show/NCT02546180 (Archived by WebCite at http://www.webcitation.org/6f6y0Dw7d).

## Introduction

**Figure 1:**
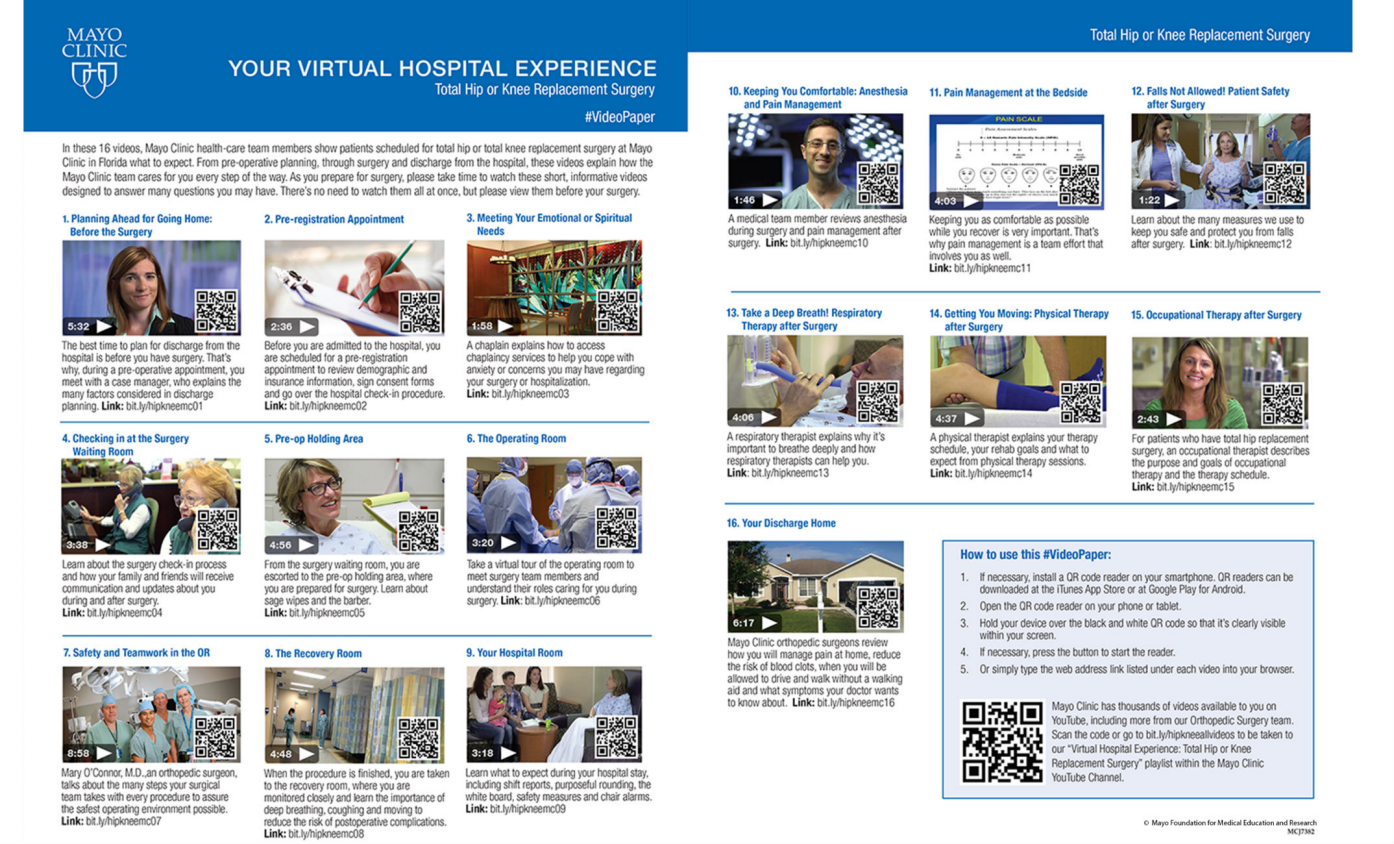
We provided the patients in the video-watching group with a handout that included the title, a brief description, and a picture, representing each video. This video sheet is our current version of the handout. The information is identical, but the images associated with each video have been altered to more accurately represent what the patient will see when accessing each video. By permission of Mayo Foundation for Medical Education and Research. All Rights Reserved.

Total knee and total hip replacement surgeries are two of the most commonly performed surgeries in patients over the age of 65. The demand and volumes for these surgeries continue to increase in accordance with the aging of the population, increasing incidence of arthritis, and patient expectations of quality of life [1]. With declining reimbursement to health care systems, face-to-face interaction between patients and providers to optimize preoperative education and counseling may be challenging. As high patient anxiety prior to surgery has been linked to more severe and persistent pain up to 1 year after joint replacement surgery, efforts to decrease patient’s concerns prior to arthroplasty may improve outcomes [2]. Recognizing the importance of preoperative education as a potential tool to decrease preoperative patient anxiety, limitations with expanding face-to-face clinical encounters, and constrained financial resources, the Orthopedic Surgery Department at Mayo Clinic Florida created a playlist of 16 YouTube videos aimed at creating a virtual hospital experience for primary total knee arthroplasty and total hip arthroplasty surgery patients. By creating a series of videos showing and explaining the anticipated hospital experience, patients would be able to virtually meet the nurses, surgeons, anesthesiologists, and others on their care team as well as view the physical locations in which their care would be delivered (eg, preoperative holding, operating room, recovery room, and hospital room; Figure 1). This method may establish a higher level of education and emotional comfort prior to surgery than with standard formats, such as pamphlets. A pilot study with patients scheduled to undergo primary hip and knee replacement surgery was then performed to evaluate usability of the video series and its potential impact on preoperative anxiety.

## Methods

Patients undergoing primary total hip or knee replacement at Mayo Clinic Florida between February and May, 2014, were asked to participate in this research study at the time of their preoperative appointment, typically 7-14 days prior to surgery. After consenting to participate, the patient completed a preoperative Generalized Anxiety Disorder (GAD) score assessment during the same appointment. The GAD score rates the patient’s self-reported level of anxiety on a scale of 0-21 (0=lowest score; 21=highest score). Patients were categorized into one of the following groups based on GAD score: minimal (GAD: 0-4), mild (GAD: 5-9), moderate (GAD: 10-14), or severe anxiety (GAD: 15-21). Patients were then randomized based on their gender, type of surgery, and initial GAD score to either the control group, which received standard education that included receiving information at routine face-to-face clinical visits and printed educational materials, or the treatment group, which included standard education plus access to the YouTube playlist. Randomization was established by the study coordinator using a preset chart with alternating treatment and control groups in each of the 16 categories (eg, a group including male, total hip replacement, minimal GAD score). On the morning of the patient’s scheduled surgery, the same surveys were repeated.

A Mayo Clinic Florida production team created the 16 videos used in this study (Figure 1). The run-time for the entire presentation was 1 hour. The videos were uploaded onto a private Mayo Clinic YouTube channel, and access to the videos required a link from the study coordinator. Patients were asked to watch all 16 videos before arriving on the morning of their scheduled surgery. In addition to watching the videos, patients were asked to rate each of the 16 videos on a scale of 1-5 points, with 1 being the lowest and 5 representing the highest level of agreement with the statement, “After watching this video I feel better prepared for surgery.” Of the 130 patients who were approached during the 3-month enrollment period, 65 agreed to participate (50% of the patients approached provided consent for participation). Those who declined enrollment cited not wanting additional information or not having enough time before their surgery date to watch videos as their reason for not participating. Of the 65 who provided consent to participate, 12 were excluded (11 for not watching at least 12 of the 16 videos and 1 for cancelled surgery). As the study progressed, new participants were randomized to appropriate treatment arms to maintain balance of the study if a participant dropped out. Of the 53 patients who participated in the study, 28 participants were in the control group and 25 in the treatment (video-watching) group (Table 1). In the treatment group, 22 patients watched all 16 videos. If patients did not watch all of the videos, it was recorded by the patients on a sheet and discussed on the morning of their surgery. Those who did not watch all of the videos cited lack of time or technical difficulties, which were quickly addressed, allowing most patients to view the videos. If patients watched less than 12 of the 16 videos, their survey answers were excluded from the dataset and the patient was withdrawn from the study. The CONSORT checklist for this study is presented in Multimedia Appendix 1. This study has been registered with clinicaltrials.gov (Registration number NCT02546180).

The distribution of patients into control and treatment groups was randomized based on gender, type of surgery (total hip arthroplasty or total knee arthroplasty), and initial GAD score (Table 1). Mean, SD, and range for the control and treatment groups are presented for age; frequency and percentage are presented for all other variables. All *P* values were calculated using Statistical Analysis Software (SAS). The *P* value for gender is based on Fisher exact test and *P* values for all other variables are based on the Kruskal-Wallis rank sum test.

**Table 1:** Patient distribution.

Characteristics		Control (n=28)	Treatment (n=25)	*P* value
**Age**				.22
	Mean (SD)	63.1 (10.7)	67.4 (10.3)	
	Range	36-78	41-86	
**Gender, n (%)**				>.99
	Males	12 (42.9)	10 (40.0)	
	Females	16 (57.1)	15 (60.0)	
**Type of surgery, n (%)**				.79
	Total knee arthroplasty	16 (57.1)	14 (56.0)	
	Total hip arthroplasty	12 (42.9)	11 (44.0)	
**Generalized Anxiety Disorder, n (%)**				.86
	Minimal (0-4)	16 (57.1)	15 (60.0)	
	Mild (5-9)	7 (25.0)	3 (12.0)	
	Moderate (10-14)	3 (10.7)	6 (24.0)	
	Severe (15+)	2 (7.1)	1 (4.0)	

## Results

Viewing this series of YouTube videos focused on creating a “virtual hospital experience” for hip and knee replacement patients may decrease preoperative anxiety. In our small series, the overall results showed a 1-point decrease in GAD score in the treatment group (Table 2). However, when the moderate and severe anxiety groups were analyzed, the declines in the median GAD score were more notable: 3 points in the control group and 6 points in the treatment group. Statistical Analysis Software (SAS) was used to run a Wilcoxon Rank sum test to determine statistical significance. None of these results reached statistical significance, as we had a low number of participants in this pilot study, particularly for the moderate to severe GAD categories. Furthermore, it is important to note that some individuals had an increase in GAD scores in both the control and treatment groups, although overall the trend showed lower GAD scores in moderate to high anxiety patients in the treatment group. Of the 12 patients with moderate to severe GAD ratings prior to surgery, 11 were women.

**Table 2:** Differences in Generalized Anxiety Disorder score.

Change in Generalized Anxiety Disorder (GAD) score	All study participants		Moderate and severe GAD scores^a^	
	Control (N=28)	Treatment (N=25)	Control (N=5)	Treatment (N=7)
Median (range)	0.0 (−7, 4)	−1.0 (−12, 7)	−3.0 (−7, 1)	−6.0 (−12, 7)

^a^11 of 12 of these patients were female.

In Table 2, the median and range of changes in GAD score from the initial to final surveys are presented for each treatment group for all study participants and for those who had moderate or severe initial GAD scores (score of 10-21). A reduction in the GAD score indicates a lower level of anxiety. The *P* values are based on Wilcoxon Rank sum tests (SAS). For all study participants, *P*=.53; for the moderate and severe GAD score group, *P*=.51.

The ratings for each video were favorable, and most participants indicated that they felt better prepared for surgery after watching the videos. Patient ratings of the individual videos ranged from 3.9 to 4.6 (1=lowest agreement; 5=highest agreement). Furthermore, the second GAD score was obtained on the day of surgery for all patients in the preoperative holding or family waiting area, providing a standard time point for assessment.

## Discussion

Medical information is easily accessed on the Internet and patients of all ages search the Internet [3]. Active Internet users include 72% of adults aged between 50 and 64 years and 41% of seniors (aged ≥ 65 years), and this audience often uses the Internet to search for health-related information [4,5]. Unfortunately, the quality of the information available on the Internet is not always exceptional. Excellence in quality and/or content of videos does not determine how many viewers a health-related video will attract; it has even been proposed that lower-quality videos receive more views [6]. By self-producing video content, medical institutions and providers can tailor accurate and appropriate educational content to their patients.

Patient recovery can be difficult to measure in a quantitative manner [7]. However, increased preoperative anxiety levels can predict worse pain outcomes in the 1st year following total knee replacement [2]. Improved preoperative education may decrease patient anxiety related to an upcoming surgical procedure. The potential benefits of reducing preoperative anxiety in patients include better postoperative recovery, higher levels of patient satisfaction with their surgical experience, and reduced levels of self-reported pain up to 1 year after surgery [2,8,9]. By providing total hip and knee replacement patients with a series of YouTube videos in an attempt to better prepare them for their surgery, they may feel better prepared for their surgical and hospital experience, decreasing anxiety and potentially improving outcomes.

Previous studies have attempted to determine the most effective means of reducing patient anxiety to improve patient recovery through the use of informational tools but have received mixed results [10-13]. Most agree that an effective means of reducing preoperative anxiety is to provide information to the patient about their upcoming hospital experience [14-16]. Various methods of delivering the information have been explored. In general, tailored information provided in a unique or interactive format yields the best results [17-19]. Educational materials for patients may be in the format of a booklet, handout or video [19]; video format permits patients to virtually meet staff members who may be involved with their postoperative care [20]. Of note, there are some patients who wish to avoid additional information and prefer to leave the details in the hands of the hospital staff [21]. These patients are referred to as “information deniers,” and by offering videos on a voluntary basis, these patients can avoid information if they do not wish to receive it [9-22]. For patients who desire more information, often referred to as “information seekers,” additional videos could be an effective means for providing this information and could result in reduced anxiety levels if the videos are of a quality that answers questions without overwhelming the patient [11,20].

Patient decision making regarding recommended surgical treatment is influenced by many factors and impacts the utilization of surgical procedures. Disparities in the utilization of surgical procedures between gender, racial, and ethnic groups have been well documented [23-25]. The patient’s willingness to proceed with surgery may be influenced by how completely their questions are answered regarding the procedure [26]. Women have been shown to ask more questions related to their upcoming surgery, have higher anxiety levels regarding their surgery, and thus may benefit more from additional information as related to reducing preoperative anxiety [11,22]. In prior research conducted by the senior author (MIO), women were shown to ask a significantly greater number of questions than men in an online preoperative patient education program for hip and knee replacement [22]. In particular, women asked questions related to their condition, the surgical procedure, and risks and benefits [22]. In this study, we also found that women had higher preoperative anxiety than men; 11 of the 12 patients with moderate to severe GAD scores in our study were women. Adequately addressing patient questions and concerns prior to surgery may require more resources for women than men.

A strength of this study is that the control and treatment groups were well balanced relative to gender, type of surgery, and preoperative GAD score (Table 2). Limitations of this study were the low numbers of patients enrolled due to time constraints of the study schedule (the resources were only available for a 3-month period). In addition, while patients were asked to record the date and number of views for each video, we did not have a method to validate the viewing of the videos by the patients. Finally, we did not study in detail whether the videos were also viewed by the patient’s family or caregivers, which could have provided additional information on the value of YouTube videos as a tool for patient and family education.

Creating innovative and effective engagement with patients to augment face-to-face encounters is essential in the new health era. With increasing financial pressure to see more patients, surgeons and their support staff may not have adequate time for addressing all concerns with each patient.

Our early experience supplementing traditional preoperative education with a series of online YouTube videos that highlight the surgical and hospital experience was well received by our patients. In our small number of patients with moderate to severe anxiety prior to surgery, we found a trend toward less anxiety after viewing the videos. Further exploration of this approach, including specific tailoring of video content to gender, race, and ethnicity may prove to be highly beneficial to both patients and health care providers.

## Appendix

Multimedia Appendix 1 CONSORT-EHEALTH checklist V1.6.2 [27].

## Abbreviations

GAD: Generalized Anxiety Disorder
