# Supplementary material for: YouTube Videos to Create a “Virtual Hospital Experience” for Hip and Knee Replacement Patients to Decrease Preoperative Anxiety: A Randomized Trial
Source: Interact J Med Res. 2016 Apr 18;5(2):e10. doi: 10.2196/ijmr.4295 (PMC4873308; doi:10.2196/ijmr.4295)
Supplement: Multimedia Appendix 1 [file jmir_v5i1e10_app1.pdf]

CONSORT 2010 checklist of information to include when reporting a randomised trial*

Section/Topic	Checklist item	Reported on page No
**Title and abstract**		
	Identification as a randomised trial in the title	1
	Structured summary of trial design, methods, results, and conclusions (for specific guidance see CONSORT for abstracts)	2
**Introduction**		
Background and objectives	Scientific background and explanation of rationale	4-5
	Specific objectives or hypotheses	4-5
**Methods**		
Trial design	Description of trial design (such as parallel, factorial) including allocation ratio	6-7
	Important changes to methods after trial commencement (such as eligibility criteria), with reasons	7-8
Participants	Eligibility criteria for participants	6-7
	Settings and locations where the data were collected	6
Interventions	The interventions for each group with sufficient details to allow replication, including how and when they were actually administered	6-8
Outcomes	Completely defined pre-specified primary and secondary outcome measures, including how and when they were assessed	6-8
	Any changes to trial outcomes after the trial commenced, with reasons	7-8
Sample size	How sample size was determined	7-8
	When applicable, explanation of any interim analyses and stopping guidelines	N/A
Randomisation:		7
Sequence generation	Method used to generate the random allocation sequence	
	Type of randomisation; details of any restriction (such as blocking and block size)	7
Allocation concealment mechanism	Mechanism used to implement the random allocation sequence (such as sequentially numbered containers), describing any steps taken to conceal the sequence until interventions were assigned	7
Implementation	Who generated the random allocation sequence, who enrolled participants, and who assigned participants to interventions	7
Blinding	If done, who was blinded after assignment to interventions (for example, participants, care providers, those assessing outcomes) and how	N/A

	If relevant, description of the similarity of interventions	7
Statistical methods	Statistical methods used to compare groups for primary and secondary outcomes	10-11
	Methods for additional analyses, such as subgroup analyses and adjusted analyses	10-11
**Results**		
Participant flow (a diagram is strongly recommended)	For each group, the numbers of participants who were randomly assigned, received intended treatment, and were analysed for the primary outcome	7-8, 12
	For each group, losses and exclusions after randomisation, together with reasons	7-8
Recruitment	Dates defining the periods of recruitment and follow-up	6
	Why the trial ended or was stopped	15
Baseline data	A table showing baseline demographic and clinical characteristics for each group	9
Numbers analysed	For each group, number of participants (denominator) included in each analysis and whether the analysis was by original assigned groups	9
Outcomes and estimation	For each primary and secondary outcome, results for each group, and the estimated effect size and its precision (such as 95% confidence interval)	11
	For binary outcomes, presentation of both absolute and relative effect sizes is recommended	N/A
Ancillary analyses	Results of any other analyses performed, including subgroup analyses and adjusted analyses, distinguishing pre-specified from exploratory	11
Harms	All important harms or unintended effects in each group (for specific guidance see CONSORT for harms)	10
**Discussion**		
Limitations	Trial limitations, addressing sources of potential bias, imprecision, and, if relevant, multiplicity of analyses	15
Generalisability	Generalisability (external validity, applicability) of the trial findings	15-16
Interpretation	Interpretation consistent with results, balancing benefits and harms, and considering other relevant evidence	15-16
**Other information**		
Registration	Registration number and name of trial registry	
Protocol	Where the full trial protocol can be accessed, if available	
Funding	Sources of funding and other support (such as supply of drugs), role of funders	

*We strongly recommend reading this statement in conjunction with the CONSORT 2010 Explanation and Elaboration for important clarifications on all the items. If relevant, we also recommend reading CONSORT extensions for cluster randomised trials, non-inferiority and equivalence trials, non-pharmacological treatments, herbal interventions, and pragmatic trials. Additional extensions are forthcoming: for those and for up to date references relevant to this checklist, see www.consort-statement.org.
